# Auditory Outcomes in Patients Undergoing Orthognathic Surgery: An Analysis of Middle Ear Disease

**DOI:** 10.1002/ohn.70022

**Published:** 2025-10-13

**Authors:** Sarah E. Hughes, Shadi Mehrabi, Grace Carvette, Anahita H. Mehta, Emily Z. Stucken

**Affiliations:** ^1^ University of Michigan Medical School Ann Arbor Michigan USA; ^2^ Department of Otolaryngology–Head and Neck Surgery University of Michigan Ann Arbor Michigan USA

**Keywords:** audiometry, conductive hearing loss, Eustachian tube dysfunction, Le Fort 1 osteotomy, Middle ear effusion, Orthognathic surgery

## Abstract

**Objective:**

To characterize postoperative Eustachian tube dysfunction (ETD) and middle ear pathology following Le Fort I osteotomy.

**Study Design:**

Retrospective cohort study.

**Setting:**

Single tertiary care center.

**Methods:**

Patients who underwent Le Fort I (LF1) osteotomy between 2000 and 2022 were identified using CPT codes. Audiometric, tympanometric, clinical, and surgical data were extracted. ETD was defined by abnormal tympanometry (type B or C), an air‐bone gap >10 dB, or PE tube placement. Patients were stratified by preoperative ETD status and classified by postoperative phenotype.

**Results:**

Among 2316 surgical patients, 151 had audiometric data. 47 patients met criteria for postoperative ETD: 15 had no prior history of ETD, 15 had a remote history of ETD, and 17 had active preoperative ETD within the year preceding Le Fort surgery. The most common phenotype of postoperative ETD was middle ear effusion (49%), followed by objective pressure abnormalities without effusion (17%), symptoms only (15%), cholesteatoma (11%), and complete Eustachian tube stenosis (9%). 28% of patients with postoperative ETD required PE tube placement and 13% required more advanced otologic surgery. Notably, 2 patients without a history of cleft palate or craniofacial syndrome developed permanent ET pathology.

**Conclusions:**

ETD may occur as a complication of Le Fort I osteotomy, even in patients without pre‐existing otologic disease.

**Level of Evidence:**

Level 4—Case series & retrospective cohort studies.

Le Fort I (LF1) osteotomy is a foundational orthognathic procedure for skeletal discrepancies including midface hypoplasia, vertical maxillary excess, malocclusion, and obstructive sleep apnea.^1^ The technique involves horizontal osteotomies of the maxilla, separating it from the nasal septum and pterygoid plates allowing controlled repositioning. Because of its proximity to the nasal cavity, maxillary sinuses, infraorbital nerves, and pterygopalatine fossa, LF1 osteotomy carries a well‐documented risk of complications such as paresthesia, infection, hemorrhage, and relapse.^2^ Auditory complications, namely the potential for Eustachian tube dysfunction (ETD) and associated middle ear pathology remain underrecognized, despite growing anecdotal concern in clinical otolaryngology practice.^2^, ^3^

The Eustachian tube (ET) regulates middle ear pressure and ventilation, supporting auditory function.^4^, ^5^ Its patency depends on the synchronized contraction of the tensor veli palatini and levator veli palatini muscles—both of which originate near the pterygoid plates and may be disrupted during LF1 osteotomy.^6^ Surgical trauma, local edema, fibrosis, or muscle detachment in this region can impair ET mechanics, resulting in negative pressure, effusion, and conductive hearing loss.^7^

Few studies have evaluated the impact of LF1 osteotomy on middle ear health and ET function.^8^ DeRuyter et al identified measurable changes in middle ear and ET function following maxillary advancement surgery.^9^ While some reports describe transient auditory changes following orthognathic surgery (eg, middle ear pressure alterations or effusion), these findings are typically based on short‐term follow‐up and small patient cohorts, relying on symptom reporting alone with limited incorporation of tympanometry or audiometry.^10^, ^11^, ^12^

While dysfunction is often assumed to be self‐limited and of minimal consequence, clinical otolaryngology practice suggests this view may be overly simplistic.^11^ There remains a paucity of data characterizing the incidence and phenotypes of ETD following LF1 osteotomy, and how this may differ in patients with or without a history of prior ETD or midface craniofacial abnormalities such as cleft palate. It is unclear whether patients without baseline ETD are also at risk for ETD following LF1.^12^

The objective of this study is to provide a comprehensive analysis of auditory outcomes in patients who underwent LF1 osteotomy, with a specific focus on both preoperative and postoperative ET function and middle ear pathology. By evaluating audiometric and tympanometric data, we aim to quantify the incidence and severity of postoperative ETD, identify potential risk factors for its development, and provide clinically relevant insights that may guide surgical decision‐making, patient counseling, and postoperative management. Given the increasing utilization of LF1 osteotomy for both functional and aesthetic indications, a deeper understanding of its impact on auditory function is critical for optimizing patient outcomes.

## Methods

### Study Design and Population

This retrospective cohort study reviewed patients who underwent LF1 osteotomy at a tertiary care hospital from January 1, 2000, to December 31, 2022. Patient records were identified using DataDirect, a clinical database query tool, using Current Procedural Terminology (CPT) and International Classification of Diseases (ICD) codes. Chart reviews confirmed surgical details and assessed audiometric tests, clinical symptoms, and physical exam findings. The study was deemed exempt by the Institutional Review Board (HUM00234753).

### Cohort Identification

2316 patients underwent LF1 osteotomy during the study period. A secondary query of an audiometric database (AudBase) yielded 151 patients from this group with at least 1 audiogram performed at the study institution. Chart reviews elicited details of their otologic history before and after surgery. At the study institution, all patients scheduled in the Otolaryngology clinic for an otologic concern are automatically scheduled for an audiogram. Patients without audiograms at our institution were presumed not to have postoperative ETD.

### Definitions

Postoperative ETD was defined by the presence of at least one of the following: type B or C tympanometry, air‐bone gap (ABG) greater than 10 dB on audiometry, persistent patient‐reported symptoms consistent with ETD without alternative diagnoses (eg, aural fullness, popping, cracking, ear pressure, muffled hearing), middle ear effusion, or placement of pressure equalization (PE) tubes following Le Fort surgery. ETD phenotypes were classified into 5 categories. The “Symptoms Only” phenotype referred to subjective aural complaints without objective audiometric abnormalities. “Pressure Abnormalities” included type C tympanograms or type B tympanograms without effusion noted on exam. “Middle Ear Effusion” was identified by a type B tympanogram with documented effusion on physical exam. “Cholesteatoma” required confirmation via otoscopy or imaging. “ET Stenosis” was defined as complete stenosis of the Eustachian tube confirmed by nasopharyngeal endoscopy or surgical findings. Diagnostic categories were independently reviewed by 2 investigators, with discrepancies resolved by consensus.

### Group Classification

Patients with audiometric data were categorized based on the presence or absence of ETD, as defined above, before and after LF1 osteotomy surgery. Of the 151 patients with available audiometric data (pre‐ or postoperative), 47 met criteria for postoperative ETD. These patients were further stratified by preoperative ETD status into 3 groups. The first group included patients with de novo ETD, defined as those with no documented history of ETD prior to surgery whose symptoms were documented to have begun postoperatively (n = 15). The second group included patients with a remote history of ETD, characterized by a prior diagnosis with no signs or symptoms of ETD within 1‐year preceding surgery (n = 15). Patients in this group generally had ETD in early childhood that had resolved prior to undergoing LF1 osteotomy. The third group included patients with active preoperative ETD, defined by symptoms or objective findings consistent with ETD within 1 year prior to LF1 surgery (n = 17).

### Data Collection

Demographic data were collected, including age at surgery, sex, race, ethnicity, body mass index, and tobacco use status. Relevant comorbidities were extracted from the medical record, including cleft palate, craniofacial syndrome, diabetes mellitus, chronic rhinosinusitis, asthma, gastroesophageal reflux disease, allergic rhinitis, chronic otitis media, and a history of PE tube placement. Surgical data included the primary indication for LF1 osteotomy (obstructive sleep apnea, midface hypoplasia or maxillary deficiency, dentofacial deformity, facial asymmetry, or posttraumatic deformity), the presence of cleft palate or craniofacial syndromes, and concurrent procedures (septoplasty, inferior turbinate reduction, distraction osteogenesis, or PE tube placement).

Audiometric evaluations were reviewed at multiple time points, including preoperative audiometry when available and up to 2 postoperative audiograms. Patients were included if they had at least 1 postoperative audiogram; not all underwent serial or longitudinal testing. Audiometric data included pure tone average thresholds for air and bone conduction, air‐bone gap, word recognition scores, and tympanometry type.^13^ The ear with the most severe findings was used for all calculations. In cases of symmetric or bilateral involvement, the right ear was used by default. Data were analyzed at the patient level to maintain clinical interpretability and avoid artificially inflating the sample size. Pre‐operative classification was based on available audiometry; otherwise, symptom documentation, exam findings, and tympanometry within the prior year were used. Patients without documentation were presumed not to have a history of ETD.

Postoperative otolaryngology clinic visit notes were reviewed for symptoms suggestive of ETD, including ear pressure, discomfort, a sensation of fullness, muffled hearing, cracking or popping sounds, tinnitus, and autophony. Otoscopic findings were recorded, including tympanic membrane retraction, middle ear effusion, tympanic membrane perforation, and cholesteatoma. Any recommended or performed interventions were documented, including medical management with nasal steroid spray with or without oral allergy medication, PE tube placement, tympanoplasty, mastoidectomy, or ET balloon dilation. When available, imaging studies, including computed tomography or magnetic resonance imaging, were reviewed for evidence of middle ear or mastoid effusion, cholesteatoma, or other abnormalities.

### Outcomes

The primary outcome was the presence of postoperative ETD following Le Fort I osteotomy. Secondary outcomes included the need for medical or surgical intervention and persistence of ETD at subsequent follow‐up.

### Statistical Analysis

Descriptive statistics characterized the frequency and clinical features of postoperative ETD. Continuous variables were reported as means with standard deviations, and categorical variables as counts and percentages. Audiometric findings were summarized for each ETD phenotype, including pure tone averages (air and bone conduction), air‐bone gap (ABG), word recognition scores (WRS), and tympanometry type. The proportion of patients with ABG > 10 dB or WRS < 90% was also reported. Descriptive analyses, including time to diagnosis and intervention, were conducted in Microsoft Excel (Version 16.78; Microsoft Corporation).

Bivariate and multivariable analyses were performed in SPSS (IBM SPSS Statistics for Windows, Version 29.0). Bivariate analyses evaluated associations between patient characteristics and postoperative ETD. Continuous variables (eg, age, BMI) were compared using Mann‐Whitney *U* tests, and categorical variables (eg, sex, race, syndromic diagnosis, preoperative ETD status) were analyzed using chi‐square or Fisher's exact tests, as appropriate.

Binary logistic regression was used to identify independent predictors of postoperative ETD. Three sequential models were constructed: (1) demographic and syndromic variables (age, sex, BMI, cleft palate, syndromic diagnosis); (2) adjustment for preoperative ETD status (de novo, remote history, active ETD); and (3) addition of clinical comorbidities (obesity, allergic rhinitis, chronic rhinosinusitis, tobacco use). Variables were selected based on biological plausibility and relevance to ETD pathophysiology. Odds ratios (ORs) with 95% confidence intervals (CIs) were reported. Statistical significance was defined as *P* < .05.

### Incidence Analysis

To evaluate the incidence of postoperative ETD, patients were grouped based on their preoperative ETD status: (1) de novo (no known history of ETD), (2) remote history (childhood ETD that had resolved), or (3) active ETD (ongoing symptoms or findings within 1 year prior to surgery).

All patients in each preoperative group were included, regardless of postoperative status. Patients with no prior history of ETD were considered de novo. Those with a past diagnosis, either remote or recent, were grouped accordingly. Incidence was calculated as the number of patients in each group who developed postoperative ETD divided by the total number of patients in that group.

Among patients with postoperative ETD, clinical phenotypes were further categorized as isolated symptoms, middle ear effusion, or permanent alterations in middle ear function, the latter defined by a diagnosis of either complete Eustachian tube stenosis or cholesteatoma. This classification described the spectrum of disease severity and identified patients at risk for persistent or irreversible dysfunction.

## Results

### Patient Cohort

A total of 2316 patients underwent LF1 osteotomy between 2000 and 2024. Audiometric data were available for 151 patients, of whom 47 met criteria for postoperative ETD. Among these, 15 had no prior ETD history (de novo), 15 had a remote history (not active within 1 year), and 17 had active ETD within 1 year prior to surgery.

The overall incidence of postoperative ETD was 2%. The incidence of postoperative ETD was 0.67% in patients with no prior history of ETD, 51.7% in those with a remote history, and 53.1% in those with active preoperative ETD.

### Demographics and Clinical Characteristics

Demographic and comorbidity profiles, stratified by ETD group, are summarized in Table 1.

**Table 1:** Demographics by ETD Group

Characteristic	No Post‐op ETD (n = 104)	Post‐op ETD (n = 47)	*P* value
Age, mean ± SD	21.3 ± 9.7	23.1 ± 11.8	.323
Sex, n (%)			.237
Male	43 (41.3%)	20 (42.6%)	
Female	61 (58.7%)	27 (57.4%)	
Race, n (%)			.744
White	91 (88.3%)	39 (83.0%)	
Black	0 (0%)	1 (2.1%)	
Asian	9 (8.7%)	5 (10.6%)	
Other/unknown	3 (2.9%)	2 (4.3%)	
BMI, mean ± SD	24.8 ± 5.7	24.6 ± 6.2	.766
Pre‐operative ETD status, n (%)			.003
None	70 (67.3%)	2 (4.3%)	
Remote history	15 (14.4%)	15 (31.9%)	
Active ETD	19 (18.3%)	30 (63.8%)	
Syndromic Diagnosis, n (%)	9 (8.7%)	12 (25.5%)	.012
Cleft Lip/Palate, n (%)	32 (30.8%)	16 (34.0%)	.654

Percentages reflect column proportions. Preoperative ETD reflects patient status prior to Le Fort I.

Abbreviation: ETD, Eustachian Tube Dysfunction.

### ETD Phenotypes

Among the 47 patients with postoperative ETD, the most common phenotype was middle ear effusion (n = 23), followed by pressure abnormalities (n = 8), symptoms only (n = 7), cholesteatoma (n = 5), and ET stenosis (n = 4). Patients with ET stenosis were found to have complete soft tissue stenosis of their ET at the torus tubarius, confirmed with rigid nasal endoscopy. All patients with ET stenosis also developed either recalcitrant middle ear effusion or cholesteatoma following LeFort osteotomy. Phenotype distributions by preoperative status are shown in Tables 2 and 3.

**Table 2:** Incidence of Postoperative ETD by Preoperative Eustachian Tube Status

Preoperative ETD status	Total patients (N)	Post‐op ETD (n)	Incidence of developing post‐op ETD (%)
No prior ETD	2255	15	0.65%
Remote history of ETD	29	15	51.7%
Active ETD < 1 year	32	17	53.1%

**Table 3:** Distribution of ETD Phenotypes Stratified by Preoperative Status

Phenotype	De Novo ETD (n = 15)	Active ETD < 1 year (n = 17)	Remote Hx ETD (n = 15)
Symptoms only	4 (26.7%)	1 (5.9%)	1 (6.7%)
Pressure abnormalities (no fluid)	3 (21.4%)	3 (17.7%)	2 (13.3%)
Middle ear effusion	6 (42.9%)	10 (58.8%)	7 (46.7%)
Cholesteatoma^a^	0 (0%)	3 (17.7%)	2 (13.3%)
ET stenosis^a^	1 (7.1%)	0 (0%)	3 (20%)

^a^ Permanent alteration.

### Audiometric Findings

Pure tone average air conduction thresholds ranged from 14.4 dB (symptoms only) to 46.9 dB (ET stenosis). Mean air‐bone gap ranged from 4.7 to 33.3 dB across phenotypes. ABG ≥ 10 dB was observed in all patients with cholesteatoma or stenosis, and in 74% of effusion, 50% of pressure abnormalities, and none with symptoms only. Tympanometry was phenotype‐specific: type A was observed in all symptoms‐only patients, type B predominated in effusion and cholesteatoma, and type C in pressure abnormalities and ET stenosis (Table 4).

**Table 4:** Audiometric, Tympanometric, and Management Characteristics by Postoperative ETD Phenotype

Phenotype	n	PTA Air (mean ± SD)	ABG (mean ± SD)	ABG > 10 dB, n (%)	WRS < 90%, n (%)	Type A (%)	Type B (%)	Type C (%)	No treatment	Medical treatment	PE tubes	Other surgery (T/M/ETD)
Symptoms only	7	14.4 ± 10.4	4.7 ± 4.5	0 (0.0%)	0 (0.0%)	100%	0%	0%	6	1	0	0
Pressure abnormalities	8	35.1 ± 16.1	19.9 ± 12.5	4 (50.0%)	0 (0.0%)	0%	0%	100%	3	1	2	2
Middle ear effusion	23	41.4 ± 21.0	26.7 ± 13.8	14 (73.7%)	0 (0.0%)	0%	84.2%	15.8%	9	3	11	0
Cholesteatoma	5	41.5 ± 10.9	33.3 ± 10.1	5 (100%)	1 (25.0%)	0%	60.0%	40.0%	0	0	1	5
ET stenosis	4	46.9 ± 21.6	32.0 ± 11.1	4 (100%)	2 (50.0%)	0%	50.0%	50.0%	0	0	0	4

Abbreviations: ABG, Air‐Bone Gap; PTA, Pure Tone Average; PE, Pressure Equalization; T/M/ETD, Tympanoplasty, Mastoidectomy, or Eustachian Tube Dilation; WRS, Word Recognition Score.

### Management Strategies

Among ETD patients, 13 (28%) received PE tubes, 5 (11%) received medical therapy, and 6 (13%) underwent advanced surgery (tympanoplasty, mastoidectomy, or ET dilation). Patients with cholesteatoma or stenosis most often required more advanced surgery. Symptoms‐only patients were primarily observed.

### Timing of Evaluation

The average time between LF1 osteotomy and postoperative audiogram among patients with ETD was 5.86 months (SD 3.84, median 5.17). Follow‐up ranged from 1 to 24 months. Among patients who developed cholesteatoma, the median time from surgery to diagnosis was 14.2 months (range: 10.3‐18.9 months).

### Multivariate Predictors of Postoperative ETD

In multivariable logistic regression adjusting for demographic and clinical factors, syndromic diagnosis was significantly associated with postoperative ETD (OR 3.66, 95% CI 1.18‐11.42; *P* = .025), while age, sex, BMI, and cleft palate were not.

Upon further adjustment for preoperative ETD status, younger age (OR 1.043 per year decrease, 95% CI 1.004‐1.084; *P* = .032), syndromic diagnosis (OR 3.47, 95% CI 1.06‐11.32; *P* = .040), and active preoperative ETD (OR 5.34, 95% CI 1.78‐16.00; *P* = .003) were independently associated with postoperative ETD. Remote ETD history was not statistically significant (OR 2.99, 95% CI 0.89‐10.05; *P* = .081).

Adding clinical comorbidities such as obesity, allergic rhinitis, chronic rhinosinusitis, and tobacco use did not significantly improve model fit or identify additional predictors. The final multivariable model demonstrated good fit (*χ*²(10) = 25.034, *P* = .005) and explained approximately 24.7% of the variance in postoperative ETD (Nagelkerke *R*² = 0.247). Full results are presented in Table 5.

**Table 5:** Predictors of Postoperative ETD on Multivariable Logistic Regression

Predictor	Odds Ratio (OR)	95% Confidence Interval	*P* value
Age (years)	1.043	1.004‐1.084	.032*
Male sex (vs female)	0.853	0.370‐1.968	.703
BMI	1.020	0.944‐1.102	.662
Cleft palate	0.804	0.276‐2.339	.696
Syndromic diagnosis	3.466	1.061‐11.319	.040*
Preoperative ETD‐Remote history (vs no ETD)	2.988	0.889‐10.048	.081
Preoperative ETD‐Active (vs no ETD)	5.342	1.783‐16.003	.003*

* Statistically significant defined at *P* < .05.

## Discussion

This study suggests that LF1 osteotomy can precipitate a spectrum of middle ear pathologies, ranging from symptomatic ear fullness to cholesteatoma and permanent ETD. While relatively uncommon, affected patients may experience chronic effusion, conductive hearing loss, or require surgical intervention. Importantly, ETD occurred not only in patients with craniofacial syndromes but also in those without prior otologic disease, challenging conventional assumptions about risk stratification.

The anatomical basis for these findings is compelling. The Eustachian tube depends on active opening by the tensor veli palatini and levator veli palatini muscles, both of which attach near the pterygoid plates—structures directly adjacent to the surgical field in LF1 osteotomy. Surgical dissection, manipulation, or fibrosis in this region can disrupt ET function, leading to impaired middle ear ventilation and negative pressure.^14^ Prior studies, such as those by DeRuyter et al and Baddour et al, have reported altered tympanometry and middle ear ventilation after maxillary advancement, while Gotzfried et al further highlighted the vulnerability of palatal musculature and Eustachian tube function to surgical disruption.^8^, ^9^, ^15^ However, these findings were limited by small cohorts and follow‐up.

This study is, to our knowledge, the first to document persistent, clinically significant de novo ETD following LF1 osteotomy in patients without syndromic diagnoses, cleft palate, or otologic history. Among these individuals, we observed a spectrum of phenotypes including effusion, pressure abnormalities, and, in 2 cases, complete postoperative stenosis of the ET resulting in lifelong otologic symptoms. In this study, persistent ETD was defined as dysfunction lasting beyond 6 months, with several patients followed for over a year. These findings expose a diagnostic blind spot: auditory symptoms in nonsyndromic patients may be misattributed or overlooked postoperatively, delaying care. Of note, each of the patients with complete postoperative ET stenosis sought the care of multiple physicians, including multiple otolaryngologists both within and outside our institution, before the diagnosis of ET stenosis was confirmed via nasopharyngoscopy within our institution. While Gleizal et al reported disabling hearing loss after Le Fort I osteotomies in patients with pre‐existing ETD, and Wong et al. described procedural variability in auditory outcomes, the de novo development of persistent ETD—including cases of both transient and permanent dysfunction—should also be recognized.^11^, ^15^

Another significant observation is the high likelihood of postoperative ETD recurrence among patients with a prior history of dysfunction. Incidence was similar among those with a remote (51.7%) or active (53.1%) preoperative history, suggesting that a childhood history of ETD—even if resolved—may confer persistent vulnerability. However, only active ETD emerged as a statistically significant predictor in multivariable regression. This may reflect limited statistical power rather than absence of effect. These findings challenge the assumption that resolved ETD carries minimal long‐term risk and underscore the importance of including both active and historical ETD in preoperative counseling. Patients with a remote history may harbor latent anatomical or functional vulnerabilities unmasked by surgical disruption, leading to recurrence.

Multivariable analyses also identified younger age and syndromic diagnosis as independent predictors of postoperative ETD. That age remained significant even after adjusting for preoperative ETD suggests an inherent physiologic or anatomic susceptibility in younger patients. In contrast, sex, BMI, and comorbidities—including obesity, allergic rhinitis, chronic rhinosinusitis, and tobacco use—were not associated with increased risk, reinforcing the specific contribution of craniofacial anatomy and surgical disruption over generalized medical comorbidity.

These findings have direct implications for clinical practice. While universal postoperative audiometric surveillance may not be feasible, the threshold for evaluation should be low, particularly in patients presenting with new‐onset aural fullness, pressure, muffled hearing, or other symptoms suggestive of ETD. Counseling should extend beyond patients with cleft palate or syndromic diagnoses to include those with resolved childhood ETD or non‐syndromic profiles. Early tympanometry and audiometry can guide timely intervention and potentially prevent progression to permanent dysfunction. Screening tools may also help identify at‐risk patients. Given the substantial morbidity associated with delayed diagnosis—including life‐long hearing loss and advanced otologic surgery—proactive patient education and monitoring protocols are warranted.

This study is limited by its retrospective nature, with inherent susceptibility to selection and ascertainment bias. Only patients who underwent audiometric evaluation were included as potentially having ETD, this may underestimate the true incidence of mild or transient ETD, or patients that developed symptoms of ETD but did not seek evaluation in our system.^16^ Additionally, reliance on clinical documentation and variable timing of audiometric testing introduces the possibility of misclassification, particularly among patients diagnosed based on symptoms alone. Because audiometric data were only available for patients evaluated within our institution, it is possible that we underestimated the true incidence of postoperative ETD, particularly in cases managed elsewhere. All patients diagnosed with postoperative ET stenosis were evaluated and diagnosed within our institution, including confirmation via nasopharyngoscopy. Nonetheless, the incorporation of objective data within a sizeable cohort strengthens the validity of the observed trends. Future prospective studies with systematic preoperative and postoperative audiometry are needed to better define the incidence of ETD, identify modifiable risk factors, and evaluate whether surgical technique modifications can reduce auditory morbidity.

This study expands the current understanding of auditory complications following LF1 osteotomy. Recognition of postoperative ETD should not be limited to syndromic or historically high‐risk populations. As the indications for orthognathic surgery continue to broaden to include patients undergoing surgery for obstructive sleep apnea, midface hypoplasia, and aesthetic indications, vigilance for middle ear sequelae are critical to optimize surgical outcomes and patient quality of life.

## Conclusion

Postoperative ETD following LF1 osteotomy is uncommon but clinically meaningful, even in patients without prior otologic history. These findings highlight the impact of surgical disruption near the Eustachian tube and underscore the need for timely symptom evaluation. Future prospective studies are needed to define incidence, identify modifiable risk factors, and develop strategies for prevention and early intervention.

## Author Contributions

**Sarah E. Hughes**, conceptualization; methodology; data curation (lead); formal analysis; writing—original draft; writing–review and editing. **Shadi Mehrabi**, conceptualization; data curation; writing—review and editing. **Grace Carvette**, Data curation; writing—original draft. **Anahita H. Mehta**, Methodology; supervision; writing—review and editing. **Emily Z. Stucken**, conceptualization; supervision (lead); project administration (lead); writing—review and editing.

## Disclosures

### Competing interests

None.

### Funding source

This study was supported by the NIH R25 grant (5R25DC20262‐02) awarded to SH through the Michigan Otolaryngology Research Education (MORE) program.
